# Supplementary material for: Unplanned Return to the Operating Room after Elective Oncologic Thoracic Surgery: A Further Quality Indicator in Surgical Oncology
Source: Cancers (Basel). 2022 Apr 20;14(9):2064. doi: 10.3390/cancers14092064 (PMC9104285; doi:10.3390/cancers14092064)
Supplement: Supplementary file 1 [file cancers-14-02064-s001.zip › cancers-1667179-supplementary.pdf]

Table S1. Patients comorbidities at first surgery by reopreation during the same admission, N (column %).

Characteristic		Same admissiom No N = 17	Same admissiom Yes N = 54	p-value
**Myocardial infarction**		1 (5.9)	3 (5.6)	1.00
**Peripheral vascular disease**		1 (5.9)	1 (1.9)	0.42
**Cerebrovascular accident or TIA**		0	2 (3.7)	1.00
**COPD**		2 (11.8)	0	0.05
**Connective tissue disease**		3 (17.7)	1 (1.9)	**0.04**
**Liver disease (Mild)**		1 (5.9)	2 (3.7)	0.57
**Chronic kidney disease**		0	1 (1.9)	1.00
**Diabetes**	**None or diet controlled**	15 (88.2)	48 (88.9)	
	**Uncomplicated**	2 (1.8)	6 (11.1)	1.00
**Solid tumor**	**Localized**	15 (88.2)	47 (87.0)	
	**Metastatic**	2 (11.8)	7 (13.0)	1.00

Table S2. Patients comorbidities at first surgery by complication, N (column %).

Characteristic		Complication: No N = 39	Complication: Yes N = 32	p-value
**Myocardial infarction**		3 (7.7)	1 (3.1)	0.62
**Peripheral vascular disease**		1 (2.6)	1 (3.1)	1.00
**Cerebrovascular accident or TIA**		1 (2.6)	1 (3.1)	1.00
**COPD**		1 (2.6)	1 (3.1)	1.00
**Connective tissue disease**		2 (5.1)	2 (6.3)	1.00
**Liver disease (Mild)**		2 (5.1)	1 (3.1)	1.00
**Chronic kidney disease**		0	1 (3.1)	0.45
**Diabetes**	**None or diet controlled**	35 (89.7)	28 (87.5)	1.00
	**Uncomplicated**	4 (10.3)	4 (12.5)	
**Solid tumor**	**Localized**	32 (82.1)	30 (93.8)	0.17
	**Metastatic**	7 (17.9)	2 (6.2)	
